# Detainee and layperson’s expectations and preferences regarding police interview rooms

**DOI:** 10.1371/journal.pone.0241683

**Published:** 2020-11-12

**Authors:** Katherine Hoogesteyn, Ewout H. Meijer, Aldert Vrij

**Affiliations:** 1 Maastricht University, Maastricht, The Netherlands; 2 University of Portsmouth, Portsmouth, United Kingdom; Baylor University, UNITED STATES

## Abstract

Emerging research on how suspects perceive the physical environment during investigative interviews yields contrasting findings. While previous studies have suggested that a room made to be physically comfortable may be optimal for interviewing suspects, another study found it can instead lead to higher suspicion of the investigator’s intentions. The current study examined current detainees’ and general population participants’ beliefs about a room that resembled a “typical” interview room, and one decorated to be warm, inviting, and comfortable. Participants also provided descriptive information about their perceptions of police interview environments (e.g., preferences, expectations). We hypothesized that the decorated room would elicit higher ratings of suspicion and wariness compared to the “typical” room. Our findings showed that, overall, participants expected to be interviewed in the “typical” room but preferred the decorated one. Contrary to our expectations, they rated the “typical” room higher on feelings of suspicion than the decorated room. The decorated room also corresponded with what participants reported to be an environment that promotes disclosure. These results bode well for conducting investigative interviews in comfortable environments.

## Introduction

Investigative interviews are vital to successful police investigations, and substantial psycholegal research focuses on the interpersonal dynamic between suspects and investigators. This interpersonal dynamic largely depends on the communication between the suspects and investigators [1], and academics have provided a plethora of recommendations for proper questioning techniques (e.g., use of open-ended, non-suggestive questions) [2], as well as for developing a constructive investigator-suspect relationship (i.e., through rapport-building) [3]. Yet, one factor of the communication process that has been overlooked thus far is the environment in which the interviews occur.

That the physical environment affects the quality of communication becomes clear from interpersonal communication research [4, 5]. For example, if a conversation takes place in a room with harsh lighting, it can lead to eyestrain or fatigue, which can then cause the communicators to feel irritable or unsettled. This, in turn, can create irritability during the conversation [4]. Further, studies from the healthcare field, for example, have found that clients’ self-disclosed more personal details when interviewed in a ‘soft’, intimate environment decorated with pictures, comfortable chairs, soft-lighting, compared to a ‘hard’, non-intimate environment characterized by block walls, uncomfortable chairs, and fluorescent lighting [6]. Similarly, Gifford [7] found that a room decorated more home-like fostered more communication concerning general and intimate topics, such as sexuality, than a more office-like decorated room. The overarching model in these studies is that comfortable, pleasant environments encourage more social interaction than sterile environments [7].

The positive findings from communication and healthcare fields may translate to an investigative interviewing context, and a few studies on the physical environment specific to investigative interviews have emerged. From examining interviews with high-value detainees, Goodman-Delahunty and Sivasubramaniam [8] identified aspects that can be strategically used by investigators to exert coercion (e.g., the use of physical restraints, isolation, and extreme temperatures) or non-coercion (e.g., soft furnishings, having refreshments available). The authors found that detainees rated their disclosure to be higher when interviewed in a comfortable environment (i.e., with non-coercive physical aspects present), noting that the comfortable environment may have fostered better rapport, which in turn facilitated [9].

Moreover, two laboratory studies–reported in Dawson and colleagues [10]–examined whether physical aspects could prime feelings of “openness” and lead to higher information disclosure in a mock-crime scenario. The “openness” manipulations included the room layout (i.e., a spacious setting), as well as décor that was metaphorically consistent with being “open” (i.e., pictures of open scenes, an open book). The interviews either took place in a larger room decorated with the openness primes, or a smaller undecorated room. Participants interviewed in the larger room provided more crime-relevant information than those interviewed in the smaller room. In one of their studies, these results were mediated by participants’ perceptions of spaciousness, that is, perceptions of greater spaciousness increased the odds of disclosure. Whereas Hoogesteyn, Meijer, and Vrij [11] did not find participants’ perceived spaciousness to influence information-disclosure regarding a mock-crime, these authors did find participants interviewed in a more spacious environment interpreted it as more physically comfortable, which in turn increased their perceptions of rapport-building. Thus, although the direct effects of a spacious environment on information disclosure seem inconclusive at the moment, at minimum, physical aspects seem to positively influence self-reported comfort and rapport-building.

A more comfortable environment may, however, also have an adverse effect on the quality of an investigative interview. In their second study, Dawson et al. [10] found that participants interviewed in the decorated room expressed higher perceptions of suspicion, decreasing information disclosure. A possible explanation for these findings is that the decorated room did not match participants’ expectations of a police interview setting, leading to suspicion of manipulation, and causing them to worry about the investigator’s suspicion against them.

The Expectancy Violations Theory (EVT) [12] could explain participants’ suspicion. EVT is an interpersonal communication theory, which posits that violations of our expectations can be positive or negative. Positive violations can elicit desirable, positive outcomes that are more advantageous than confirmations, while negative violations can elicit undesirable reactions, of less advantage than a confirmation. According to the EVT, individuals use these expectations to inform their perceptions and frame their interactions with others [12]. In Dawson and colleagues’ case, if interviewees were exposed to a room that was ‘nicer’ (i.e., decorated) than what they expected, a negative expectancy violation could explain the higher suspicion and the decreased odds of disclosure following from that.

### The present study

Determining how police interview environment are perceived by suspects can provide insight into how interview rooms should be designed. Because perceptions of suspicion can be counter-productive to communication [13], we were interested in examining whether Dawson et al.’s [10] results would replicate–that is, if individuals would report a comfortably decorated interview room to elicit higher feelings of suspicion. To examine this, we asked participants to compare two rooms, one resembling a “typical” interview room, and one decorated to be warm, inviting, and comfortable. We predicted that participants would expect to be interviewing in the “typical” room and, stemming from Dawson et al.’s [10] findings, would rate the decorated room higher on suspicion compared to the “typical” room.

Further, we were interested in an additional exploration of what individuals’ expectations are of what police interview rooms look like, and to also gather their interview room preferences.

We collected data from two groups–the general population as well as from current detainees. We gathered responses from current detainees because they are the most representative of the ‘target’ individual during investigative interviews [14]. While majority of the research on interview strategies have relied on police investigator’s data [15–17] few studies have examined detainees’ perspectives (see 9, 14, for exceptions). Gathering information from the target population is essential, as cooperation is ultimately the suspect’s decision, researchers must then also examine what suspects think of the interview to obtain a more complete picture of what occurs in the interview room [14].

## Methods

### Participants and procedure

This study was approved by Maastricht University's Ethical Review Committee Psychology and Neuroscience (ERCPN). All data and materials are available through the Open Science Framework (OSF; https://osf.io/fbkmw/).

#### Detainees

Our sample consisted of 78 detainees. Their age range was 17 to 69 years (*M* = 32.15 years, *SD* = 13.52) and the majority were male (58 men, 19 women, 1 preferred not to say). To gauge the level of familiarity detainees may already have with police interview environments, we asked them whether they had been interviewed by the police in previous occasions. Forty-four, out of the 78, reported that they had, and of those who provided the reason for the interviews, sixteen were due to theft, nine due to drug-related charges, six due to violating a restraining order, two due to quarreling, one for driving without a license, one for scamming, and one for being an accomplice.

All detainees were recruited from one detainee center in Amsterdam (NL). The term ‘detainee’ refers to individuals who have been arrested and are placed in custody for a set period of time, pending further development in their case (e.g., an official interview, a hearing, displacement to jail). The recruitment of the detainees took place over a three month period, in which a research assistant from the Dutch Police Academy approached incoming detainees’ cells and asked if they were willing to participate in a research study. It was made clear to all detainees that participation was strictly voluntary and unrelated to the detainee center, at no cost or benefit to them. The research assistant first provided detainees with an explanation of the study and asked for consent that was obtained verbally. Waiver of documentation of consent was required as detainees were not allowed to hold writing utensils due to safety precautions, and approved by the ethical committee to further protect anonymity. After consenting, the research assistant first asked the detainee how they were doing that day and proceeded to provide each question verbally while writing down the responses. Upon completion, the detainees were given the chance to drop-out, and if choosing so, their responses would be discarded immediately. Lastly, they were provided with an email address on a paper note in case they had any questions or concerns and were thanked for their participation.

#### General population

We gathered a hundred and one responses through Amazon Mechanical Turk (MTurk), where the study was advertised as looking for people’s thoughts regarding police interview rooms in exchange for 1USD. Twenty-two responses had to be excluded due to not properly responding to the open-ended questions, either by entering numbers or random sentences that were not consistent with the prompted question. Thus, our final general population sample consisted of 79 participants. Their age range was 20 to 58 years (*M* = 31.57 years, *SD* = 8.94), the majority were male (49 men, 30 women). Before the survey started, we asked participants whether they had been previously questioned by police; those who said yes were excluded from participating. Our exclusion criteria were pre-registered through the OSF.

#### Survey

**Fig 1:**
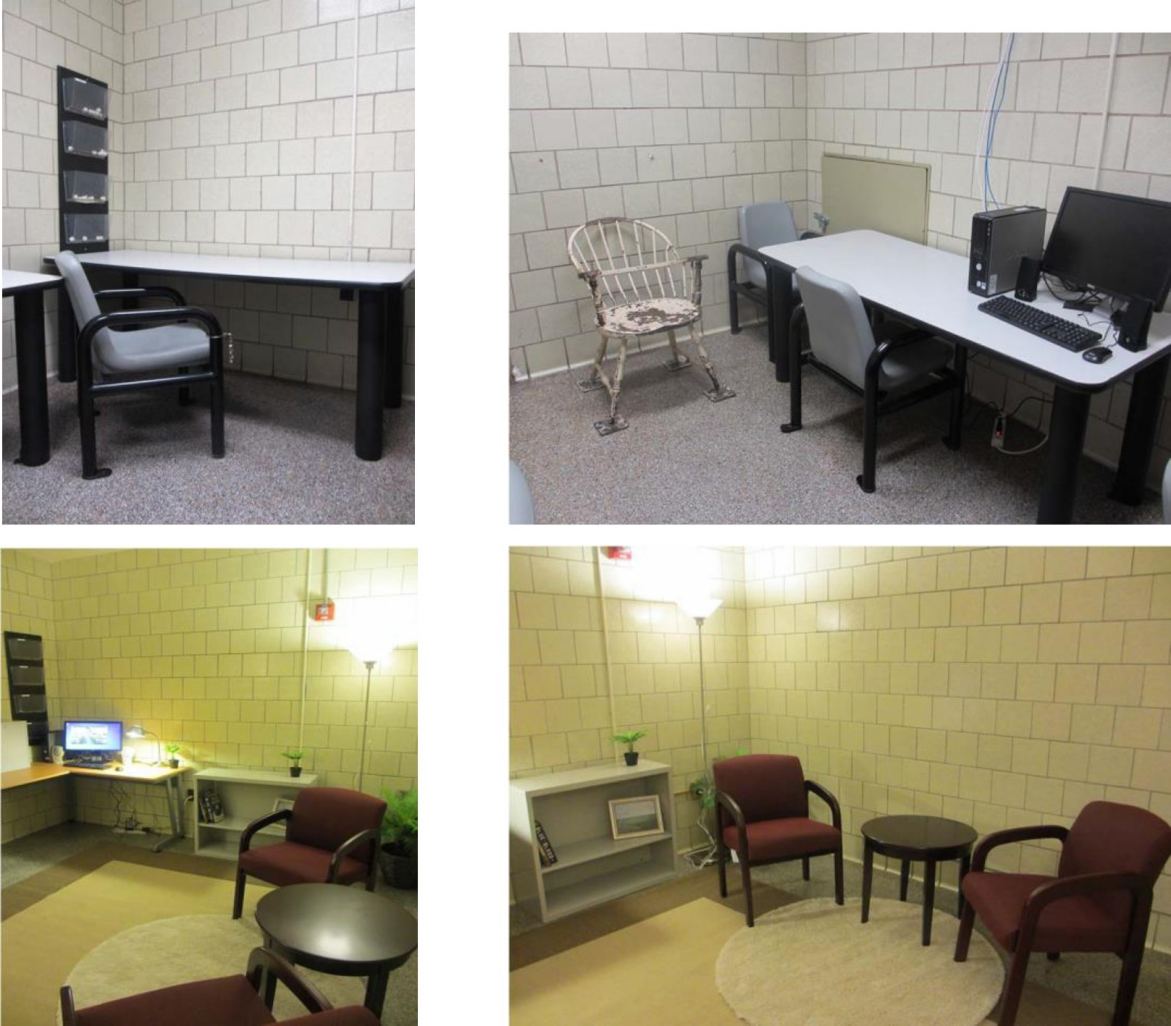
Images of typical room (top) and decorated room (bottom).

The questionnaire comprised six questions (see S1 Appendix for full questionnaire). All questions were phrased through the lens of a suspect scenario. Two open-ended questions gathered 1) what participants’ expectations were of suspect interview locations, and 2) what they thought this location should look like in order to promote disclosure. We then presented participants with photos of two nearly identical rooms of the same size. These photos were provided to us by Kelly and colleagues [18] from the Southwest Detectives Division of the Philadelphia Police Department (U.S.A). One of the photos depicted an interview room in its current form, with no decorations, fluorescent lighting and uncomfortable chairs (which we refer to from now on as the “typical” room; see Fig 1). The other photo depicted a second room that was altered and decorated in order to make the space more inviting, comfortable, and warm by including office-like decorations, soft lighting, and comfortable chairs (we refer to this room as the “decorated” room; see Fig 1).

For each room, participants were asked to indicate how it made them feel from a selection of 3) seven positive (i.e., comfortable, able to speak freely, cooperative) and 4) negative (i.e., suspicious, constrained, ready to get out, wary) characteristics, presented via 7-point Likert-type scales (1 = *not at all* to 7 = *extremely)*. The presentation of the two room photos was counterbalanced. Participants were then 5) asked to choose which of the two rooms most accurately represented what they expected a suspect interview room to look like as well as 6) which room they, as suspects to a crime, would prefer to be interviewed in, providing descriptive explanations for why.

We then averaged the seven characteristics into composite ratings, determined by both face-validity and high correlational values. “Suspicious” and “wary” were combined to form an overall suspicion score (*r* = .645, *p* < .001), “able to speak freely” and “cooperative” were combined to form an overall cooperation score (*r* = .736, *p* < .001), “constrained” an “ready to get out” were combined to form an overall constraint score (*r* = .543, *p* < .001), we left “comfortable” on its own, as this is a more general characteristic (see Table 1 for all correlation values).

**Table 1:** Intercorrelations (Pearson’s r) between all seven room characteristics.

	Comfortable	Suspicious	Constrained	Able to speak freely	Cooperative	Ready to get out	Wary
Comfortable	-	-.374**	-.447**	.548**	.478**	-.493**	-.332**
Suspicious		-	.467**	-.352**	-.284**	.400**	.645**
Constrained			-	-.379**	-.300**	.543**	.411**
Able to speak freely				-	.736**	-.394**	-.377**
Cooperative					-	-.362**	-.376**
Ready to get out						-	.329**
Wary							-

Note.

** *p*  < .001.

#### Coding

All open-ended, unstructured responses to room expectations and preferences were coded into data-derived categories that best represented the data. The first author went through each of the responses identifying several underlying themes and created dominant categories per question, all overlapping responses were combined and condensed into the dominant categories. For example, the question on what the interview setting should look like to promote disclosure yielded 14 categories, including that the room should be open, that it should be inviting, and should have comfortable chairs.

To establish inter-rater reliability, the main coder (the first author) and a second independent coder coded a randomly selected 20% of the participants’ open-ended responses into the appropriate categories, achieving acceptable reliability (see S1 Table for the exact values). All coding data are available on the OSF (https://osf.io/fbkmw/).

## Results

The detainee sample was subject to omissions, particularly for the open-ended questions. Therefore, the number of categories endorsed do not equal the sample size. All percentages represent the proportion of respondents who answered the question as opposed to the whole sample, the number of total respondents to that question (*N*) is noted next to all percentages.

### Interview room expectations

We asked participants to select which of the two rooms (i.e., the decorated or typical one) they would expect to be interviewed in as suspects to a crime. A majority of participants, from both groups, expected the typical room over the decorated one, and a comparison of the proportions between detainees and the general population revealed no significant difference between their expectations (*X*^2^ (1, *N* = 148) = 2.7, *p* = .113).

Of the detainee sample, 72.5% (*N* = 69) selected the typical room over the decorated one, and 60 provided some open-ended response as to why. The most reported explanation for expecting the typical room was that the decorated one looked too comfortable to be a suspect interview setting (*n* = 18), followed by that the typical room simply reflected what an actual interview room looks like (*n* = 14), and that the typical room looked more authoritarian, stricter, or colder (*n* = 2). Of those participants who selected expecting to be interviewed in the decorated room (27.5%, *N* = 69) the most cited reason was that the typical room looked old and outdated (*n* = 11). A less reported reason for expecting the decorated room was that it was more spacious than the typical room (*n* = 2).

Among the general population group, 83.5% (*N* = 79) reported expecting to be interviewed in the typical room, with the most provided reasons being that it resembled what they see on television (*n* = 21), that the decorated room looked too comfortable to be a suspect interview room (*n* = 17), and that the typical room reflected authority, strictness, or the coldness associated with suspect interviews (*n* = 17). Of those that reported expecting to be interviewed in the decorated room (16.5%, *N =* 79), the most reported reason was that it looked more comfortable and humane (*n* = 7). As one participant wrote: *“[the decorated room] is more comfortable*. *When you investigate anyone*, *to try to speak freely*, *you don't scare him*… . *[the decorated room] looks like home*, *then they will speak freely and coordinate to you”*.

### Interview room ratings

To examine the effect of room photo stimuli (typical room or decorated room) on the four subjective ratings, and to account for the interdependence between each participant’s repeated measures, we conducted a series of linear mixed models in SPSS version 26. Group (detainee or general population) and room (typical room or decorated room) as well as their interaction terms were examined as fixed factors. We also controlled for detainees’ previous interview experience (yes or no) and gender. The models were estimated using a compound symmetry structure and maximum likelihood estimation. Table 2 presents the results from the linear mixed models and Table 3 presents the marginal means for each variable level.

**Table 2:** Linear mixed model for suspicion, cooperation, constraint, and comfort.

	*Fixed effects*	*Estimate (SE)*	*t(df)*	*p-value*	*95% CI*
***Suspicion***					
	Intercept	4.95(.27)	18.29(197.5)	< .001	[4.42, 5.49]
	Group	-1.23(.27)	-4.53(245.5)	< .001	[-1.76, -0.69]
	Room	0.96(.19)	5.00(155.4)	< .001	[0.58, 1.34]
	Interviewed before	-0.55(.27)	-2.02(157.8)	.044	[-1.08, -0.01]
	Gender	-0.63(.20)	-0.31(154.6)	.757	[-0.47, 0.34]
	Group* Room	0.69(.26)	2.60(154)	.010	[0.16, 1.22]
***Cooperation***					
	Intercept	4.12(.30)	13.32(180.9)	< .001	[3.51, 4.73]
	Group	-0.74(.31)	-2.37(214.5)	.019	[-1.36, -0.12]
	Room	-0.56(.19)	-2.82(146.8)	.005	[-0.95, -0.16]
	Interviewed before	1.24(.31)	3.93(149.4)	< .001	[0.62, 1.87]
	Gender	0.004(.24)	0.02(148.2)	.984	[-0.47, 0.48]
	Group* Room	-1.17(.27)	-4.30(146.7)	< .001	[-1.71, -0.63]
***Constraint***					
	Intercept	3.23(.30)	10.60(191.2)	< .001	[2.63, 3.83]
	Group	0.55(.31)	1.77(231.8)	.078	[-0.06, 1.17]
	Room	1.45(.21)	6.62(149.6)	< .001	[1.01, 1.88]
	Interviewed before	-0.45(.30)	-1.45(151.7)	.148	[-1.06, 0.16]
	Gender	-0.08(.23)	-0.36(149.2)	.717	[-0.54, 0.37]
	Group* Room	0.53(.29)	1.78(148.2)	.076	[-0.05, 1.11]
***Comfort***					
	Intercept	5.30(.28)	18.70(217.1)	< .001	[4.74, 5.86]
	Group	-0.67(.30)	-2.22(268.1)	.027	[-1.26, -0.07]
	Room	-3.08(.24)	-12.53(152.9)	< .001	[-3.56, -2.59]
	Interviewed before	-0.20(.28)	-0.72(154.2)	.469	[-0.76, 0.35]
	Gender	0.28(.21)	1.35(151.5)	.176	[-0.13, 0.70]
	Group* Room	1.04(.33)	3.07(151.9)	.003	[0.37, 1.71]

**Note**. Factors were dummy coded for Group as: Gen. Pop (0) and Detainee (1); Room as: Typical (0) and Decorated (1); Interviewed before as No (0) and Yes (1); Gender as Male (0) and Female (1). t-tests used Satterthwaite approximated degrees of freedom.

**Table 3:** Marginal means, standard error, and 95% confidence interval for ratings of suspicion, cooperation, constraint, and comfort.

	Suspicion	95% CI	Cooperation	95% CI	Constraint	95% CI	Comfort	95% CI
**Group**								
Gen. Pop	4.24(.19)	[3.86, 4.62]	3.13(.22)	[2.70, 3.56]	4.51(.21)	[4.09, 4.93]	3.65(.19)	[3.26, 4.04]
Detainee	5.13(.14)	[4.84, 5.41]	4.46(.17)	[4.12, 4.80]	3.69(.16)	[3.36, 4.02]	3.80(.15)	[3.51, 4.10]
**Room**								
Typical	5.34(.13)	[5.07, 5.61]	3.22(.15)	[2.92, 3.52]	4.96(.15)	[4.66, 5.26]	2.45(.14)	[2.15, 2.74]
Decorated	4.03(.13)	[3.76, 4.30]	4.37(.15)	[4.07, 4.68]	3.24(.15)	[2.94, 3.55]	5.01(.14)	[4.71, 5.30]
**Interviewed before**								
Yes	4.96(.22)	[4.51, 5.41]	3.17(.26)	[2.66, 3.68]	4.33(.25)	[3.59, 4.16]	3.83(.23)	[3.37, 4.28]
No	4.41(.12)	[4.17, 4.65]	4.42(.14)	[4.13, 4.71]	3.88(.14)	[3.83, 4.82]	3.62(.12)	[3.37, 3.88]
Group*Room								
Gen. Pop. / Typical Room	5.07(.21)	[4.65, 5.49]	2.26(.23)	[1.79, 2.73]	5.50(.23)	[5.04, 5.97]	2.63(.22)	[2.18, 3.08]
Gen. Pop./ Decorated Room	3.41(.21)	[2.99, 3.83]	4.00(.23)	[3.53, 4.47]	3.52(.23)	[3.06, 3.99]	4.67(.22)	[4.22, 5.12]
Detainee / Typical Room	5.56(.17)	[5.27, 5.94]	4.18(.20)	[3.79, 4.57]	4.41(.20)	[4.02, 4.81]	2.26(.19)	[1.88, 2.64]
Detainee / Decorated Room	4.64(.17)	[4.31, 4.98]	4.74(.20)	[4.35, 5.14]	2.96(.20)	[2.57, 3.36]	5.34(.19)	[4.96, 5.72]

**Note.** All ratings were measured on a 1 (not at all) to 7 (extremely) Likert-type scale.

There was a statistically significant main effect of room on suspicion that was not in the hypothesized direction. Contrary to our hypothesis, both detainees and the general population rated the typical room higher on suspicion compared to the decorated room. There was a statistically significant group*room interaction. Examination of the marginal means indicated that the detainee group rated both rooms higher on suspicion compared to the corresponding ratings by general population participants.

We also found statistically significant main effects of room on cooperation, constraint, and comfort, with the overall sample rating the decorated room higher on feelings of comfort and cooperation, and lower on feelings of constraint, compared to the typical room.

Regarding cooperation, there was also a significant group*room interaction, so that, regardless of room, the detainee group rated feelings of cooperation higher than the general population. Notably, detainee’s previous interview experience had a significant main effect on reported feelings of cooperation, so that previously interviewed detainees reported lower cooperation levels than those who had yet to go through an official police interview, although we note that the marginal means for cooperation were overall moderate.

### Interview room preference

Overall, majority of participants reported preferring to be interviewed in the decorated room over the typical one, with no significant difference between groups on their preferences (*X*^2^ (1, *N* = 149) = 4.0, *p* = .067).

Most of the detainees reported preferring to be interviewed in the decorated room as suspects to a crime (98.6%, *N* = 70), and 62 detainees provided some open-ended response as to why. The most reported reasons were that the decorated room was warmer or nicer (*n* = 20), as well as more comfortable (*n* = 13), and would put them more at ease (*n* = 6) than the “typical” room. Other less cited reasons for preferring the decorated room was that it looked home-like (*n* = 4), more humane (*n* = 2), and more spacious (*n* = 2) than the “typical” room. The one participant that selected preferring the “typical” room (1.4%, *N* = 70) did not provide an open-ended response as to why.

Similarly, 91.1% (*N* = 79) of the general population reported preferring the decorated room. Out of the reasons reported, the most cited were feelings of higher comfort (*n* = 33), more ease (*n* = 20), more open to talk (*n* = 17), more personable or inviting (*n* = 14), and warmer or nicer (*n* = 14) than the “typical room”. Other less cited reasons for preferring the decorated room were that it was more humane (*n* = 4), less suspicious (*n* = 2), and home-like (*n* = 2) than the “typical” room. For example, one participant stated: *“There is already a high base level of anxiety involved in being questioned by police officers*. *I don't want to be subjected to an environment that accentuates that feeling of anxiety any further*, *because most likely I am innocent and the last thing I want to do is give them a reason to suspect otherwise”*.

Out of the 8.9% (*N* = 79) of general population participants who selected preferring the “typical” room over the decorated one, three participants stated that the “typical” room looked more to the point, as, for example, a participant stated: “*The [typical room]*, *does not pretend to be something it is not”*.

### What do people think interview settings look like?

We also asked participants to describe what they thought a police interview setting looks like through an open-ended prompt. From the detainees who provided some open-ended response (*N* = 60), the most reported related to furniture (i.e., amount of chairs, table present, and computers; *n* = 34), followed by the interview room being bare or unadorned (*n* = 7), resembling an office (*n* = 7), or small in size (*n* = 2).

From the general population (*N* = 79), the most reported responses also related to the interview room furniture (*n* = 45), being a bare or unadorned room (*n* = 38), small in size (*n* = 18), dark (*n* = 16), having a two-way mirror (*n* = 15), florescent lighting (*n* = 9), gray (*n* = 8), windowless (*n* = 7), having uncomfortable chairs (*n* = 6), resembling what they see on television (*n* = 6), cold (*n* = 4), and with concrete floors or walls (*n* = 4). Lastly, some of the general population reported the interview room as an intimidating setting (*n* = 3)–as one participant described: “*Cold*, *empty*, *not much to look at*. *Not very comforting*. *A prison cell without the bars”*.

### What should the interview setting look like to promote disclosure?

Participants were asked to report on what they thought an interview setting should look like to promote disclosure through an open-ended, descriptive prompt. Out of the 52 detainees that provided responses, the most reported answer was that the rooms were fine as they currently are (*n* = 12), others responded that the rooms should have some color or decoration (*n* = 9), should have items such as coffee, water, or snacks available (*n* = 4), and should have windows (*n* = 3. Moreover, some detainees reported that the room simply did not matter to them (*n* = 3).

From the general population group (*N* = 79), the most reported characteristic that a room should have was to be comfortable or relaxing (*n* = 23), bright (*n* = 16), have comfortable chairs (*n* = 14), and some color or decoration (*n* = 12). Other responses included that the rooms should resemble an office or homey space (*n* = 9), have windows (*n* = 8), be spacious (*n* = 7), and overall should be inviting (*n* = 7). For example, one participant stated: *“I think the room should be more inviting per se*. *Not everyone being interviewed is necessarily guilty of a crime*, *so I don't feel that it's right to have them in an intimidating environment*. *People would probably talk more if they were treated like less of a criminal”*. Conversely, some participants reported that the rooms should look authoritarian or sterile (*n* = 7).

## Discussion

In this study, we examined the beliefs of detainee and general population individuals on two different police interview environments, one “typical” room and one designed to be more comfortable and inviting. Detainee and general population participants overall expected to be interviewed in the typical room, as opposed to the decorated one. However, against our expectation, the decorated room did not elicit higher suspicion or wariness compared to the typical room.

Our results contrast Dawson and colleagues’ [10] suggestion that, in their study, the decorated room elicited higher suspicion because it may have violated participants’ expectations. While we found that participants thought the decorated room appeared more comfortable than they would expect for a suspect interview room, this expectancy violation was positive. That is, the unexpected room environment was interpreted as a favorable environment. The decorated room corresponded with what the majority of participants described qualitatively to be an environment that promotes disclosure, which should be relaxing, include comfortable chairs, decorations, and appear home-like. Such positive expectancy violations are promising, as the EVT posits that a violation triumphs a confirmation of an expectation, as long as it is a positive violation [12]. Since the decorated room did not elicit higher suspicion, we encourage academics and practitioners to closely examine how a more physically comfortable interview room could facilitate information disclosure.

The finding that both groups preferred to be interviewed in the decorated room fits with recent interest in determining what constitutes effective police interview environments [9, 19] and our qualitative data provides insight into what such an environment may be. Participants indicated that interview rooms should be made more comfortable, including a general population participant who indicated, “*I’d be more open to speaking in a generally non-threatening location that is warm and promotes civil conversation”* Notably, recent data also indicates that police investigators support making interview environments more comfortable and less sterile [20], suggesting that some current interviewing contexts should be improved. Beyond perceptions of comfort, emerging research also suggests that detainee disclosure may be enhanced in a more physically comfortable environment [9]. Therefore, a more comprehensive examination of suspects’ disclosure in different interviewing contexts is warranted.

We also found that both detainees and lay people rated the decorated room as eliciting higher feelings of cooperation, supporting that the interview room’s environment could influence suspects’ cooperation efforts, echoing Goodman‐Delahunty and colleagues’ [9] findings. The higher feelings of cooperation together with the lower feelings of suspicion can be promising for positively influencing the dynamic between investigators and suspects. Research has found that that individuals who feel like they are suspects tend to hold more negative views about the police’s legitimacy, and lower legitimacy views are also linked to lower cooperation with the legal system [21]. However, this must be interpreted with caution as we relied on self-reports. Future research could incorporate other forms of objective data, such as the actual amount of information disclosure, combined with a lens model analysis to better assess how self-reported levels of cooperation predict actual cooperation.

Of note, previous interview experience influenced detainees’ cooperation ratings, so that those who had been interviewed before provided overall lower ratings. Similarly, Snook and colleagues’ [22] found that detainees’ self-reported levels of cooperation were lower when they had previous experience with the criminal justice system compared to those who had not. It is possible that their (possibly negative) previous experience, and the circumstances of findings themselves again in police custody, makes them cautious, even distrustful, of reporting on cooperation. Alternatively, this finding could reflect that repeat offenders are less likely to cooperate in general.

We found that the general population participants expected to be interviewed in a “typical” room partly because it reflected the authority, strictness, or the coldness associated with suspect interviews. One participant stated, *“I would expect police to treat suspects to an environment where they are not too comfortable and to keep them on the edge to incite mistakes in interview”*. An explanation for this expectation could be the participants associating the criminal justice system with punishment. Our general population group mostly came from the United States, where public opinion on criminal justice matters is known to be punitive [23]. None of the detainees, who came from the Netherlands, reported such reasons for expecting the “typical” interview room. Besides the situational differences between the general population and detainees, there could be underlying distinctions in public opinions regarding the criminal justice system between Americans and the Dutch. It would be interesting for future research to delve into cross-cultural differences in opinions pertaining to criminal justice matters and how these may guide individuals’ perceptions of investigative interviewing environments.

Notably, the images of the decorated room we showed participants was manipulated by Kelly and colleagues [18] in many ways, including the lighting, the furniture and its arrangement, as well as the décor. While our results bode well for conducting investigative interviews in comfortable environments, it would be useful, particularly for informing police agencies, for future research to establish which specific room manipulations are essential to create a comfortable environment. Based on our findings, for example, detainees and the general population mentioned the use of colors and decorations. In Kelly and colleagues’ [18] study, the decorated room included several decorations, however, the room was used to conduct witness interviews, while in our study we focused on suspect scenarios. Some decorations may be more or less appropriate depending on who the interviewee is, especially considering safety precautions.

Another direction for future studies is to more closely examine investigators’ thoughts about interview environments. While in our study we focused on suspects’ expectations and preferences, investigative interviews are dynamic and bi-directional interactions. It is possible that a decorated room negatively violates the expectations of investigators, depending on the interview room they are accustomed to conducting their practice in. Future studies should also account for how investigators perceive the environment, and whether this influences their behavior (see 18).

This study was subject to limitations. First, our design was analogous to a vignette study, asking participants to rate and compare two sets of interview room’s photos. While this design made it feasible to obtain data from the detainee population, vignette studies limit the level of involvedness participants may feel [24]. Additionally, the data collection method from our two samples differed. Detainees were asked the questions in person for no compensation at the detention center, while MTurkers completed the questionnaire online and with a monetary compensation. For the detainee group, this procedure could have resulted in a biased sample. While all incoming detainees were approached at the detention center for interest in participation, we had no control over the characteristics of the detainees who agreed to participate and those who chose not to engage.

There are also limitations to data collection via MTurk. Specific to our study, the main concerns dealt with participants’ motivation and attention. To counteract these concerns, we ensured our compensation amount was higher than the average for comparable studies, while also keeping our survey as succinct as possible. We were also careful with the responses we allowed in the final sample, checking all open-ended responses for signs of bots, inattention, and poor quality. Moreover, the quality of MTurk data compared to data collected from other methods, such as telephone surveys, is supported by previous studies [25–27]. In terms of advantages, our MTurk data had relatively lower missing data, while detainees provided less detailed responses and had higher omission rates. However, the difference in response rates may be due to the current situation of the detainees, as well as other individual variables that we did not account for (e.g., lack of sleep, mental illness).

To conclude, we found that detainees and general population individuals expected a police interview setting to resemble a “typical” room–that is, including the bare minimum (i.e., a table, chairs) and to be simple, sterile and undecorated. Yet, they reported preferring a room to be decorated, warm, and comfortable in order to create a disclosing environment. Against our expectations, and previous findings by Dawson and colleagues [10], being presented with a decorated, as opposed to a “typical” room, did not appear to negatively violate participants’ expectations of a suspect interview room. Rather, we found that the expectancy violation was positive. Thus, future studies should examine how a more nicely decorated, physically comfortable, environment may be useful for facilitating the suspect-investigator relationship as well as eliciting information. For example, in this survey, participants mentioned that decorations, colors, comfortable chairs, and windows are aspects that can help create a disclosing environment. These alterations are feasible and largely under the control of practitioners [9] and can offer implications for when planning the interviews and (re)designing police interview rooms.

## Supporting information

S1 Appendix (DOCX)

S1 Data (SAV)

S2 Data (SAV)

S1 Table Reliability statistics for qualitative coding of open-ended responses. (DOCX)

## References

[1] Yeschke CL. The art of investigative interviewing: A human approach to testimonial evidence. Butterworth-Heinemann; 2003.

[2] Clarke C, Milne R. A national evaluation of the PEACE Investigative Interviewing Course. London: Home office; 2001.

[3] Abbe A, Brandon SE. The role of rapport in investigative interviewing: A review. Journal of investigative psychology and offender profiling. 2013 10;10(3):237–49.

[4] Hartley P. Interpersonal communication. Routledge; 2002 1 4.

[5] Knapp ML, Hall JA, Horgan TG. Nonverbal communication in human interaction. Cengage Learning. 2013.

[6] Chaikin AL, Derlega VJ, Miller SJ. Effects of room environment on self-disclosure in a counseling analogue. Journal of Counseling Psychology. 1976 9;23(5):479.

[7] Gifford R. Light, decor, arousal, comfort and communication. Journal of environmental psychology. 1988 9 1;8(3):177–89.

[8] Goodman-Delahunty J, Sivasubramaniam D. Investigative and intelligence interviewing in Asia-Pacific jurisdictions.Report to the US Department of Justice, Federal Bureau of Investigation and Centre for Law and Human Behaviour University of Texas, El Paso. Manly: Charles Sturt University 2013.

[9] Goodman‐Delahunty J, Martschuk N, Dhami MK. Interviewing high value detainees: Securing cooperation and disclosures. Applied cognitive psychology. 2014 11;28(6):883–97.

[10] Dawson E, Hartwig M, Brimbal L, Denisenkov P. A room with a view: Setting influences information disclosure in investigative interviews. Law and human behavior. 2017 8;41(4):333 10.1037/lhb0000244 28459264

[11] Hoogesteyn K, Meijer E, Vrij A. The influence of room spaciousness on investigative interviews. Legal and Criminological Psychology. 2019 9;24(2):215–28.

[12] Burgoon JK. Expectancy violations theory. The International Encyclopedia of Interpersonal Communication. 2009.

[13] Burgoon JK, Buller DB, Ebesu AS, White CH, Rockwell PA. Testing interpersonal deception theory: Effects of suspicion on communication behaviors and perceptions. Communication Theory. 1996 (6): 243–267.

[14] Cleary HM, Bull R. Jail inmates’ perspectives on police interrogation. Psychology, Crime & Law. 2019 2 7;25(2):157–70.

[15] Kassin SM, Leo RA, Meissner CA, Richman KD, Colwell LH, Leach AM, et al Police interviewing and interrogation: A self-report survey of police practices and beliefs. Law and human behavior. 2007 8 1;31(4):381–400. 10.1007/s10979-006-9073-5 17253153

[16] Kelly CE, Redlich AD, Miller JC. Examining the meso-level domains of the interrogation taxonomy. Psychology, Public Policy, and Law. 2015 5;21(2):179.

[17] Miller JC, Redlich AD, Kelly CE. Accusatorial and information-gathering interview and interrogation methods: a multi-country comparison. Psychology, Crime & Law. 2018 10 21;24(9):935–56.

[18] Kelly CE, Dawson E, Hartwig M. Context manipulation in police interviews: a field experiment. Journal of Experimental Criminology. 2019 12 17:1–20.

[19] Meissner CA, Kelly CE, Woestehoff SA. Improving the effectiveness of suspect interrogations. Annual review of law and social science. 2015 11 3;11:211–33.

[20] Hoogesteyn K, Meijer E, Vrij A. Utility and effectiveness of the context manipulation techniques: police investigators’ perspectives. Journal of Police and Criminal Psychology. 2020 3 4:1–8.

[21] Tyler TR, Jackson J, Mentovich A. The consequences of being an object of suspicion: Potential pitfalls of proactive police contact. Journal of Empirical Legal Studies. 2015 12;12(4):602–36.

[22] Snook B, Brooks D, Bull R. A lesson on interrogations from detainees: predicting self-reported confessions and cooperation. Criminal Justice and Behavior. 2015 (42):1243–1260.

[23] Cullen FT, Fisher BS, Applegate BK. Public opinion about punishment and corrections. Crime and justice. 2000 1 1;27:1–79.

[24] Hughes R, Huby M. The construction and interpretation of vignettes in social research. Social Work and Social Sciences Review. 2012 12 26;11(1):36–51.

[25] Crump MJ, McDonnell JV, Gureckis TM. Evaluating Amazon's Mechanical Turk as a tool for experimental behavioral research. PloS one. 2013 3 13;8(3):e57410 10.1371/journal.pone.0057410 23516406 PMC3596391

[26] Fox J, Rooney MC. The Dark Triad and trait self-objectification as predictors of men’s use and self-presentation behaviors on social networking sites. Personality and Individual Differences. 2015 4 1;76:161–5.

[27] Kees J, Berry C, Burton S, Sheehan K. An analysis of data quality: Professional panels, student subject pools, and Amazon's Mechanical Turk. Journal of Advertising. 2017 1 2;46(1):141–55.

