# Supplementary material for: Detainee and layperson’s expectations and preferences regarding police interview rooms
Source: PLoS One. 2020 Nov 12;15(11):e0241683. doi: 10.1371/journal.pone.0241683 (PMC7660492; doi:10.1371/journal.pone.0241683)
Supplement: S1 Appendix — (DOCX) [file pone.0241683.s001.docx]

**S1 Appendix**

**Detainee Survey**

Hello, my name is [researcher’s name], and I am a PhD student at [university] and [university] I am conducting a study to better understand how people feel about police interview rooms, and I invite you to complete the short questionnaire that was provided to you. I am interested in your perceptions and expectations regarding interview rooms. This questionnaire should take approximately 7 minutes to complete, and your complete and honest responses would be very informative regarding police practice and the investigative interview process. Please keep in mind that the questionnaire is designed to preserve your anonymity. In other words, your individual responses will not be shared with the police, or anyone other than me, the experimenter. At the end of the study, your responses will be combined with others who also participated and will be reported as averages in our publication.

Thank you very much for your attention; your thoughts regarding interview rooms are valuable and I am deeply appreciative for the information you provide.

**Demographics:**

Age: _____

Gender: _____

Have you been officially interviewed by the police before?

If so: Day ___ Month ___ Year ___

For what purposes were you last interviewed by police? _______________

In what location were you last interviewed by police? ________________

Have you been admitted to prison before?

If so: Day ___ Month ___ Year ___

For what offenses were you last admitted to prison? ________________

The following questions will ask about your opinions about police interview rooms. Please read the questions carefully, and answer thoroughly and honestly.

1. Can you please describe in your own words what you **expect** a suspect interview location to look like?

_____________________________________________________________

_____________________________________________________________

_____________________________________________________________

_____________________________________________________________

1. Can you please describe in your own words how you think a suspect interview location **should look like** in order to encourage you to be talkative?

_____________________________________________________________

_____________________________________________________________

_____________________________________________________________

_____________________________________________________________

Take a careful look at the following photos of Room A^[[1]](#footnote-1)^

**ROOM A:**

1. If you were interviewed in ROOM A. How would you feel? Please answer all that apply:
   1. Comfortable

| 1  Not at all | 2 | 3 | 4  Somewhat | 5 | 6 | 7  Extremely |
| --- | --- | --- | --- | --- | --- | --- |

- 1. Suspicious

| 1  Not at all | 2 | 3 | 4  Somewhat | 5 | 6 | 7  Extremely |
| --- | --- | --- | --- | --- | --- | --- |

- 1. Constrained

| 1  Not at all | 2 | 3 | 4  Somewhat | 5 | 6 | 7  Extremely |
| --- | --- | --- | --- | --- | --- | --- |

- 1. Able to speak freely

| 1  Not at all | 2 | 3 | 4  Somewhat | 5 | 6 | 7  Extremely |
| --- | --- | --- | --- | --- | --- | --- |

- 1. Cooperative

| 1  Not at all | 2 | 3 | 4  Somewhat | 5 | 6 | 7  Extremely |
| --- | --- | --- | --- | --- | --- | --- |

- 1. Ready to get out

| 1  Not at all | 2 | 3 | 4  Somewhat | 5 | 6 | 7  Extremely |
| --- | --- | --- | --- | --- | --- | --- |

- 1. Wary

| 1  Not at all | 2 | 3 | 4  Somewhat | 5 | 6 | 7  Extremely |
| --- | --- | --- | --- | --- | --- | --- |

Take a careful look at the following photos of Room B

**ROOM B:**

1. If you were interviewed in ROOM B. How would you feel? Please answer all that apply
   1. Comfortable

| 1  Not at all | 2 | 3 | 4  Somewhat | 5 | 6 | 7  Extremely |
| --- | --- | --- | --- | --- | --- | --- |

- 1. Suspicious

| 1  Not at all | 2 | 3 | 4  Somewhat | 5 | 6 | 7  Extremely |
| --- | --- | --- | --- | --- | --- | --- |

- 1. Constrained

| 1  Not at all | 2 | 3 | 4  Somewhat | 5 | 6 | 7  Extremely |
| --- | --- | --- | --- | --- | --- | --- |

- 1. Able to speak freely

| 1  Not at all | 2 | 3 | 4  Somewhat | 5 | 6 | 7  Extremely |
| --- | --- | --- | --- | --- | --- | --- |

- 1. Cooperative

| 1  Not at all | 2 | 3 | 4  Somewhat | 5 | 6 | 7  Extremely |
| --- | --- | --- | --- | --- | --- | --- |

- 1. Ready to get out

| 1  Not at all | 2 | 3 | 4  Somewhat | 5 | 6 | 7  Extremely |
| --- | --- | --- | --- | --- | --- | --- |

- 1. Wary

| 1  Not at all | 2 | 3 | 4  Somewhat | 5 | 6 | 7  Extremely |
| --- | --- | --- | --- | --- | --- | --- |

1. In which room would you **expect** to be interviewed in as a suspect to a crime? Please circle one.

Room A

Room B

Please explain why:

_____________________________________________________________

_____________________________________________________________

_____________________________________________________________

_____________________________________________________________

1. In which room would you **prefer** to be interviewed in as a suspect to a crime? Please circle one.

Room A

Room B

Please explain why:

_____________________________________________________________

_____________________________________________________________

_____________________________________________________________

_____________________________________________________________

Thank you for taking the time to complete this questionnaire. Your responses will be kept confidential, and no report resulting from this data will linked to you.

1. Presentation of Room A and Room B were counter-balanced [↑](#footnote-ref-1)
