# Supplementary material for: Detainee and layperson’s expectations and preferences regarding police interview rooms
Source: PLoS One. 2020 Nov 12;15(11):e0241683. doi: 10.1371/journal.pone.0241683 (PMC7660492; doi:10.1371/journal.pone.0241683)
Supplement: S1 Table — (DOCX) [file pone.0241683.s004.docx]

| **S1 Table** | | | | |
| --- | --- | --- | --- | --- |
| *Reliability statistics for qualitative coding of open-ended responses* | | | | |
| Question | Category | Cohen’s Kappa | Approximate Significance | Percentage  Agreement |
| **Think room looks like** | Intimidating | -- | -- | 100 |
|  | Gray | 1.00 | < .001 | 100 |
|  | Concrete | 0.66 | < .001 | 97.5 |
|  | Windowless | -- | -- | 100 |
|  | With a two-way mirror | 1.00 | < .001 | 100 |
|  | Bare | 0.78 | < .001 | 92.5 |
|  | Parent or lawyer present | -- | -- | 100 |
|  | Angry police present | -- | -- | 100 |
|  | Table/chairs/computers present | 0.79 | < .001 | 90 |
|  | Uncomfortable chairs | 1.00 | < .001 | 100 |
|  | Small | 0.68 | < .001 | 92.5 |
|  | Like an office | 1.00 | < .001 | 100 |
|  | Cold | -- | -- | 100 |
|  | Fluorescent lighting | 0.66 | < .001 | 97.5 |
|  | Dark | 0.84 | < .001 | 97.5 |
|  | Like in television | 0.66 | < .001 | 97.5 |
| **What room should look like** | Open | 1.00 | < .001 | 100 |
|  | Fine as is | 0.63 | < .001 | 90 |
|  | Does not matter | -- | -- | 100 |
|  | Have a window | 0.84 | < .001 | 95 |
|  | Coffee present | 0.72 | < .001 | 92.5 |
|  | Authoritative | -- | -- | 100 |
|  | Private | -- | -- | 100 |
|  | Should be nicer | 0.79 | < .001 | 97.4 |
|  | Decorations/color | 0.92 | < .001 | 97.4 |
|  | Comfortable chairs | 0.62 | < .001 | 92.3 |
|  | Bright | 0.68 | < .001 | 92.3 |
|  | Comfortable/relaxing | 0.68 | < .001 | 92.3 |
|  | Inviting | 0.72 | < .001 | 94.8 |
|  | Like a living room/office | 1.00 | < .001 | 100 |
| **Expect typical room, why?** | Colder/stricter than decorated room | 0.68 | < .001 | 84.6 |
|  | Decorated room is too comfortable | 0.93 | < .001 | 97.2 |
|  | Decorated room looks like a living room | -- | -- | 100 |
|  | It is how they are | 0.80 | < .001 | 94 |
|  | Looks like in television | 0.84 | < .001 | 97.2 |
| **Expect decorated room, why?** | It is larger | -- | -- | 100 |
|  | It is humane | -- | -- | 100 |
|  | The typical room looks old | -- | -- | 100 |
| **Reasons for preferring the decorated room** | It is more comfortable | 0.71 | < .001 | 86.1 |
|  | It is warmer/nicer | 0.81 | < .001 | 91.7 |
|  | Feel more at ease | 0.72 | < .001 | 94 |
|  | It is more personable | 0.68 | < .001 | 91.7 |
|  | Feel more open to talking | 0.72 | < .001 | 94 |
|  | Looks like a living room | -- | -- | 100 |
|  | It is more humane | -- | -- | 100 |
|  | It is more spacious | -- | -- | 100 |
|  | It is less suspicious | 1.00 | < .001 | 100 |
| **Reasons for preferring the typical room** |  |  |  |  |
|  | It is “to the point” | -- | -- | 100 |
| Note. The reliability values are computed on the raw data from both raters. Both raters agreed on the complete absence of 15 codes and the Cohen’s Kappa was not informative as both raters’ scores were constant values (labeled as --). Additionally, some of the categories’ marginal distributions were extremely skewed, resulting in relatively lower Kappa values even though the percentage agreements were consistently high. | | | | |
